# *Saussurea involucrata SiLEA5* Enhances Tolerance to Drought Stress in *Solanum lycopersicum*

**DOI:** 10.3390/foods13223641

**Published:** 2024-11-15

**Authors:** Xiaoyan Liu, Aowei Li, Guanghong Luo, Jianbo Zhu

**Affiliations:** 1 Gansu Engineering Technology Research Center for Microalgae, Hexi University, Zhangye 734000, China; liuduo@stu.shzu.edu.cn; 2 College of Life Sciences, Shihezi University, Shihezi 832000, China; liaowei241@mails.ucas.ac.cn

**Keywords:** ABA, DHN, drought resistance, stomatal density, *P5CS*

## Abstract

Drought adversely affects plant growth, which leads to reduced crop yields and exacerbates food insecurity. Late embryogenesis abundant (LEA) proteins are crucial for plants’ responses to abiotic stresses. This research further investigates the role of *SiLEA5* by utilizing transgenic tomatoes under drought stress. The expression of *SiLEA5* was upregulated under drought and abscisic acid (ABA) treatment, resulting in decreased electrolyte leakage and malondialdehyde content, alongside increased levels of osmotic regulators and antioxidant enzyme activity. These biochemical alterations reduce oxidative damage and enhance drought resistance. qRT-PCR analysis revealed the upregulation of ABA signaling genes and key enzymes involved in proline biosynthesis (P5CS) and dehydrin (DHN) synthesis under drought stress. Additionally, overexpression of *SiLEA5* increased the net photosynthetic rate (Pn) and fruit yield of tomatoes by regulating stomatal density and aperture. These findings suggest that *SiLEA5* may be a potential target for improving drought tolerance in tomatoes and other crops.

## 1. Introduction

Global climate change has increased the frequency of natural disasters, with abiotic stresses such as drought, high temperatures, and low temperatures severely affecting plant populations [1,2]. Drought is considered the most severe of these stresses, disrupting plant water balance and significantly impacting growth and development. *Solanum lycopersicum*, an economically important crop widely used in cooking and the food industry [3], has an annual production of approximately 18.02 million tons [4]. However, drought poses serious challenges to tomato cultivation, impacting both yield and quality, especially in arid regions like Xinjiang, China, where the dry, semi-arid climate places considerable pressure on agricultural productivity [5]. Investigating tomato responses to drought stress and understanding their adaptive mechanisms is, therefore, essential.

Late embryogenesis abundant proteins (LEA), abundant in plant tissues, are structurally flexible and play a critical role in managing plant stress responses, attracting significant research interest [6]. These proteins are categorized into eight subfamilies based on conserved structural domains, with varying numbers in different species. For example, *Arabidopsis* contains 51 distinct LEA proteins [7], while *Capsicum annuum* has 82 [8]. Under stress conditions, certain LEA proteins undergo conformational changes that help maintain cellular hydration, stabilize protein structures, and enhance plant stress tolerance [9]. The overexpression of *SiLEA14* significantly improves salt and osmotic stress resilience in transgenic *Arabidopsis* and *Setaria italica* while also promoting Escherichia coli growth [10]. Furthermore, LEA proteins enhance drought resistance in *Glycine max* [11] and *Triticum aestivum* [12] by modulating leaf gas exchange and reactive oxygen species, underscoring their role in regulating plant thermotolerance.

Plants exposed to abiotic stresses such as drought, high salinity, and low temperatures rely on abscisic acid (ABA) as a key hormone that regulates stress responses [13]. ABA activates specific signal transduction pathways to enhance tolerance. Among these pathways, LEA proteins have received significant attention for their crucial role in protecting cells from dehydration and oxidative stress [14,15]. Studies show that LEA proteins are highly expressed during seed development and in response to abiotic stress, with the ABA-responsive element (ABRE) significantly overexpressed in LEA gene promoters, suggesting that ABA signaling was critical for LEA protein regulation [16]. Under stress conditions like drought, ABA levels in plants increase, triggering a series of ABA signal transduction pathways [17]. Consequently, LEA proteins are key effectors in the ABA signaling pathway, contributing to plant stress responses.

Previous research demonstrated that LEA proteins could significantly enhance the cold tolerance of tomatoes [18]. However, there are few reports on the response of *SiLEA* proteins to drought. This study focuses on the mechanism of action of *SiLEA5* from *Saussurea involucrata* under drought stress. These findings provide new insights into the genetic breeding of tomato varieties with improved drought resistance.

## 2. Materials and Methods

### 2.1. Isolation and Promoter Analysis of SiLEA5 Gene

The *SiLEA5* gene, isolated from *Saussurea involucrata*, was described in detail in our previous studies [19]. The recombinant plasmid pCAMBIA2300-*SiLEA5*, which was preserved in our laboratory, was utilized in this study. We selected the 2000 bp sequence upstream of the ATG transcription start site of the *SiLEA5* gene as promoter sequence, and the prediction of cis-acting elements was completed using the PlantCARE online website at(https://bioinformatics.psb.ugent.be/webtools/plantcare/html/, accessed on 5 March 2024).

### 2.2. Plant Material and Growth Conditions

The wild-type tomato variety was “Yaxin 87-5”, provided by Yaxin Seed Co., Ltd. (Shihezi, China). In this study, the T2 generation seeds of SiLEA5 transgenic tomatoes were obtained from previous research conducted by our group and have been stored in the laboratory [19]. The seeds are planted at an internal temperature of 28 °C, a relative humidity of 40–60%, and 16 h of light/8 h of darkness. The light intensity is set to 80 µmol/m^2^/s. The ratio of red light spectrum energy (655–665 nm) to far-red light spectrum energy (725–735 nm) is maintained between 1.0 and 1.2. All plants are grown under professional LED growth lights from Philips, the Netherlands, ensuring the consistency of lighting conditions and repeatability of experiments.

### 2.3. Drought Stress Treatment

The drought stress experiment was conducted indoors. Wild-type and transgenic tomato seeds with uniform size and plumpness were selected, soaked in distilled water for 12 h, and then sown in flowerpots filled with a mixed cultivation medium (peat soil: vermiculite: perlite = 3:1:2, *v*/*v*). The flowerpots were covered with plastic film and placed in a cultivation room. After 15 days, the seeds had germinated, and the plastic film was removed. At 40 days post-germination, tomato seedlings with uniform growth were selected for the drought stress experiment. The experimental design included two treatment groups and one control group, with each group containing nine seedlings and three replicates. At day 0, all plants were fully watered. In the treatment groups, watering was withheld to apply simulated drought stress, while the control group continued regular watering [20]. After the drought stress period, all plants were rewatered for a 7-day recovery phase. Morphological changes were observed and recorded at regular intervals throughout this experiment. Samples were collected on day 0, day 7 (moderate stress), and day 20 (severe stress) for physiological and biochemical analyses.

### 2.4. Growth Status and Biochemical Analysis

The relative growth rate (RGR) of plants, leaf relative water content, and leaf water loss rate were determined using the gravimetric method [21]. Root vitality in tomatoes was assessed using the TTC method [22]. Relative conductivity was measured with an EC 215 conductivity meter (Markson Science Inc., Del Mar, CA, USA). Malondialdehyde content was quantified using the TBA method [23]. The activities of the enzymes CAT, SOD, POD, and APX were determined using colorimetric assays [24]. In brief, 0.5 g of leaf samples were homogenized in a mortar with PBS buffer on ice to obtain crude extracts. The resulting homogenates were mixed with the respective reagents to create reaction mixtures, and absorbance values were measured at different wavelengths. Osmoregulatory substances, including soluble sugars, soluble proteins, and proline, were determined using commercial assay kits obtained from Sangon Biotech Co., Ltd. (Shanghai, China). All chemicals used in this study were purchased from Solarbio Science & Technology Co., Ltd. (Beijing, China). All measurements were performed with three biological replicates.

### 2.5. Stomatal Density and Shape

Wild-type and transgenic tomato leaves under normal growth, drought stress, and 100 µm ABA treatment were selected to make 0.1–0.5 mm thick slices. The observation was performed using an optical microscope (CH20BIMF200; Olympus Optical Co., Ltd., Tokyo, Japan). We employed a manual counting method to determine the stomatal frequency within each visual field. Additionally, the morphometric parameters of stomatal size and aperture were subjected to quantitative analysis using ImageJ v1.8.0 software.

### 2.6. Photosynthetic Physiology and Yield Analysis

In the experimental field (44°20′ N, 85°30′ E, Shihezi, China), we cultivated 15 wild-type and 15 transgenic lines for data collection. The planting area covered 10.5 square meters, with a plant spacing of 30 cm and a row spacing of 50 cm. Throughout this experiment, we adhered to standard agricultural management practices, including regular irrigation based on crop growth conditions, appropriate fertilization, and timely pest and disease control to minimize damage. Photosynthesis-related parameters were quantified using a LI-6400XT portable gas exchange photosynthesis system (LICOR Biosciences, Lincoln, NE, USA). Detailed measurement protocols can be found in the operation manual (https://www.licor.com/env/support/LI-6400/manuals.html, accessed on 5 March 2024). The following photosynthetic parameters were measured: intercellular CO_2_ concentration (Ci); net photosynthesis rate (Pn); stomatal conductance (Gs); transpiration rate (Tr); maximum photochemical efficiency of Photosystem II (Fv/Fm); non-photochemical quenching (Npq); and photochemical quenching coefficient (qp). All measurements were performed between 9:00 and 11:00 AM on sunny days. Water use efficiency (WUE) was calculated as the ratio of Pn to Tr. Plant height was measured using a tape measure to determine the distance from the ground to the fifth spike. Stem thickness was assessed by measuring the stem circumference 1.00 cm above ground level with a vernier caliper. The internode length was measured with a tape measure from the leaves between the second and third panicles. For fruit count per plant, all fruits from the first to fifth clusters were collected and tallied. Fruit weight was determined using a precision analytical balance. Transverse and longitudinal fruit diameters were measured with vernier calipers, and the fruit shape index was calculated as the ratio of the longitudinal diameter to the transverse diameter. Soluble solids were evaluated using a handheld sugar meter. Six plants were randomly selected for testing, and three fruits from the second and third clusters of each plant were sampled for analysis. All data analyses were based on mean values.

### 2.7. Gene Expression Analysis

Total RNA was extracted from tomato leaves using a plant total RNA extraction kit. First-strand cDNA was synthesized using an inversion library kit (Takara Biotechnology, Kusatsu, Japan). qRT-PCR was performed in a 20 μL reaction using the Roche LightCycler^®^ 480 system and SYBR Green Real-Time PCR Master Mix (Roche, Salt Lake City, UT, USA; KAPA Biosystems, Wilmington, DE, USA). The amplification procedures were as follows: 95 °C for 5 min; 95 °C for 30 s; 60 °C for 30 s; 72 °C for 30 s, 25 cycles; 72 °C for 10 min. *GAPDH* was used as the housekeeping gene for normalization [25]. All primer sequences are provided in Supplementary Materials (Table S1). This experiment included three independent biological replicates, and fold changes were calculated using the 2^−ΔΔCt^ method.

### 2.8. Statistical Analysis

All measurement data were subjected to at least three biological replicates, and statistical analysis was completed with IBM SPSS Statistics 27 (SPSS Inc., Chicago, IL, USA) through analysis of Student’s *t*-test. Statistical graphics were plotted using GraphPad Prism version 8.3 for Windows (GraphPad Software, San Diego, CA, USA, www.graphpad.com). * *p* < 0.05, ** *p* < 0.01, *** *p* < 0.001, and **** *p* < 0.0001.

## 3. Results

### 3.1. Bioinformatics Analysis of SiLEA5

Using the PlantCARE online tool, we predicted the cis-acting elements within the 2000 bp upstream promoter sequence of the *SiLEA5* gene (Table 1). The results indicated that the promoter region contains core promoter elements, including the TATA-box, CAAT-box, and GATA-box. Additionally, it features various stress-responsive and hormone-responsive elements associated with growth hormone, gibberellin, abscisic acid (ABA), and jasmonic acid responses. Notably, there are two ABA response components, ABRE (ACGTG), located at 1537 bp and 1561 bp. These findings suggest that *SiLEA5* may be involved in multiple processes, including plant growth, development, and responses to abiotic stress.

### 3.2. SiLEA5 Promotes Tomato Growth and Improves Tomato Drought Resistance

**Figure 1:**
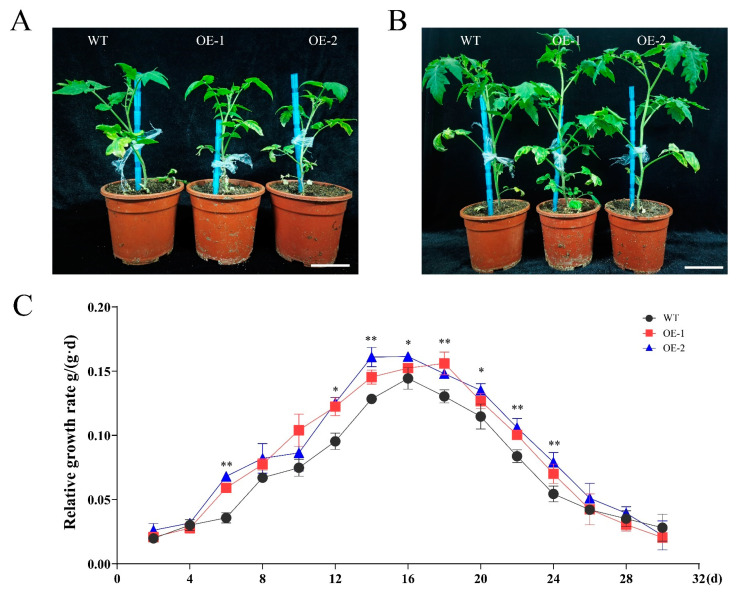
Phenotypes of wild-type and transgenic tomato under normal growth. (**A**,**B**) Growth phenotypes of wild-type and transgenic tomato. (**C**) Relative growth rate (RGR). Data are means ± SD of three replicates. (* *p* < 0.05, and ** *p* < 0.01 for comparisons between the transgenic lines and wild-type plants by Student’s *t*-tests). Bar = 3 cm.

Through phenotypic observation, we found that the plant height and overall size of the transgenic lines were significantly greater than those of the wild-type plants (Figure 1A). Relative Growth Rate (RGR) analysis during the vegetative growth stage indicated a progressive increase in RGR, with the wild-type reaching a peak RGR of 1.4 g/(g·d) at day 46, while the transgenic lines (OE-2) achieved a higher peak RGR of 1.6 g/(g·d) at day 44. This suggests that the transgenic lines exhibit a faster growth rate during the vegetative stage, potentially attributable to the introduction of the *SiLEA5* gene, which may enhance growth efficiency. Further analysis during the reproductive growth stage revealed a deceleration in RGR across all lines, likely due to the plants beginning to allocate resources towards reproductive structures such as flowers and fruit development. Nonetheless, the transgenic lines maintained a significantly higher RGR than the wild-type throughout both the vegetative and subsequent reproductive growth stages, demonstrating their superior growth capacity.

**Figure 2:**
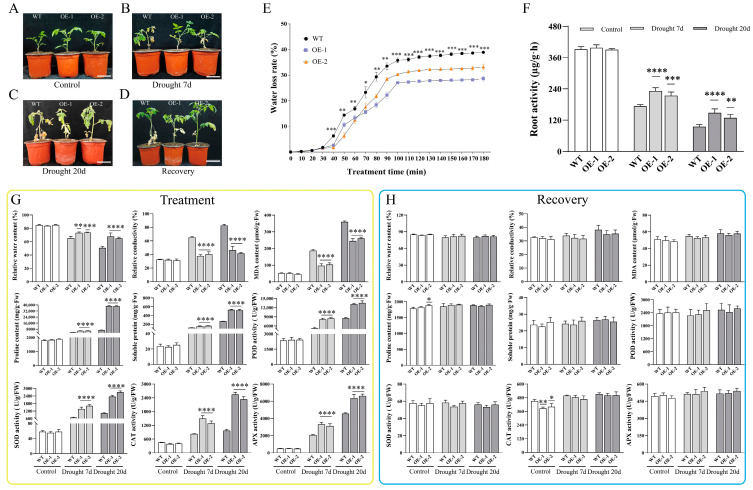
Biochemical analysis of tomato under drought stress. (**A**–**D**) Growth phenotype of tomato. (**E**) Changes in leaf water loss rate. (**F**) Determination of root activity. (**G**) Biochemical changes under different drought stress levels. (**H**) Biochemical changes after rehydration. Data are means ± SD of three replicates. (* *p* < 0.05, ** *p* < 0.01, *** *p* < 0.001, and **** *p* < 0.0001 for comparisons between the transgenic lines and wild-type plants by Student’s *t*-tests). Bar = 8 cm.

Before drought treatment, both wild-type and transgenic lines exhibited similar healthy and vigorous growth. After 20 days of drought stress, wild-type showed severe damage, including leaf wilting, curling, yellowing, and stunted growth. In contrast, transgenic lines maintained normal growth. Following rehydration, transgenic lines recovered quickly, while wild-type only partially survived (Figure 2A–D). The water loss rate in transgenic leaves was lower than in wild-type, indicating better water balance (Figure 2E). Drought stress also reduced root activity in wild-type (Figure 2F), hindering water and nutrient uptake. Additionally, drought increased osmotic mediators like proline, which helps maintain cell osmotic stability, and elevated antioxidant enzyme activities, which protected cells from oxidative damage. Transgenic lines showed higher performance in these aspects compared to wild-type plants (Figure 2G). After rehydration, physiological and biochemical indices returned to normal, indicating restored drought tolerance in tomatoes (Figure 2H). In conclusion, *SiLEA5*-overexpression improved the drought resilience of tomatoes.

### 3.3. SiLEA5 Enhances Photosynthetic Capacity by Regulating Stomatal Changes

**Figure 3:**
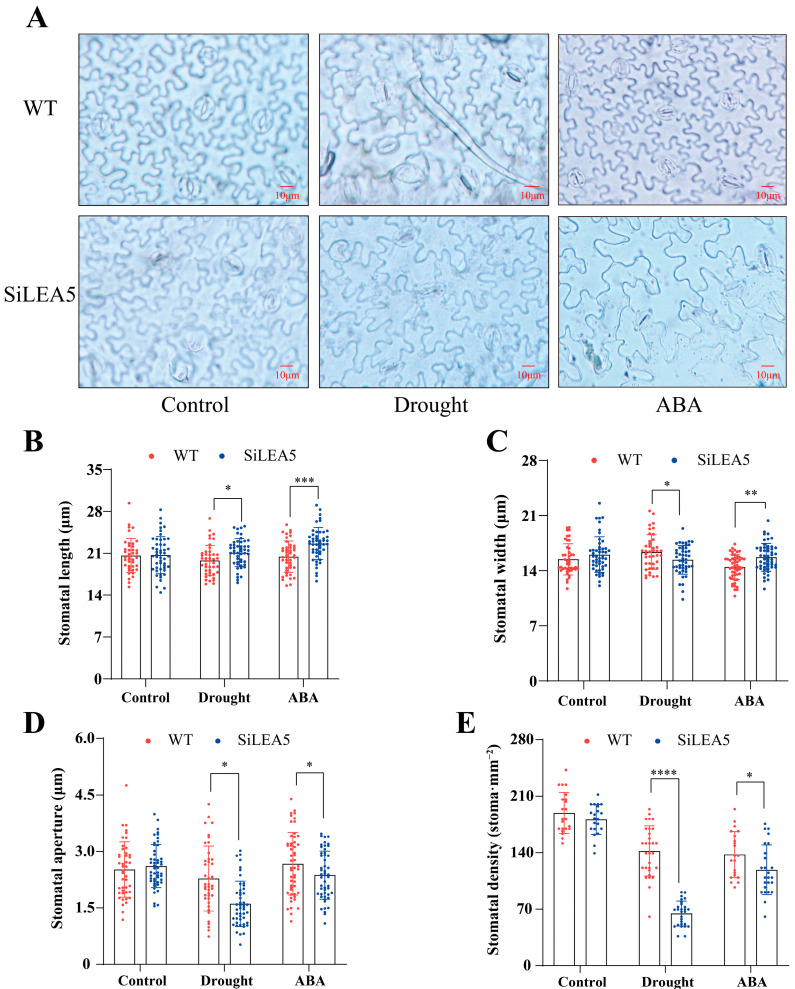
*SiLEA5* increases stomatal sensitivity to drought stress and ABA. (**A**) Stomatal changes in tomato under drought stress and ABA treatment. (**B**,**C**) Stomatal length and width (µm). (**D**) Stomatal aperture (µm). (**E**) Stomatal density (stoma·mm^−2^). Error bars, mean ± SD. The asterisks indicate a statistically significant difference (two-tailed Student’s *t*-test, * *p* < 0.05, and ** *p* < 0.01, *** *p* < 0.001, and **** *p* < 0.0001).

ABA, as a key signaling molecule that induces stomatal closure, plays a central role in plant drought response. In order to explore the role of *SiLEA5* in this process and its potential association with the ABA signaling pathway, we investigated stomatal pore size changes in tomato leaves under drought and ABA treatment. As shown in Figure 3, compared with the wild-type, the stomatal opening and stomatal density of *SiLEA5*-overexpressed tomatoes significantly decreased under drought and ABA treatment, which did not occur under control conditions. These findings suggest that the *SiLEA5* gene may enhance the adaptability of transgenic tomatoes to drought by regulating stomatal behavior. In addition, the *SiLEA5* gene may play an important role in ABA-mediated stomatal regulation.

**Figure 4:**
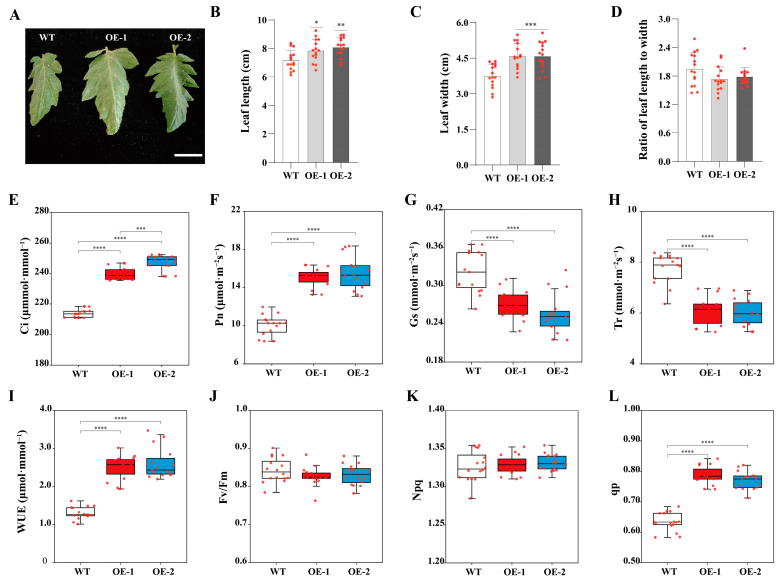
Analysis of photosynthetic capacity of wild-type and transgenic tomatoes under drought stress. (**A**) Leaf phenotype. (**B**) Leaf length. (**C**) Leaf width. (**D**) Ratio of leaf length to width. (**E**) Intercellular CO_2_ concentration (Ci). (**F**) Net photosynthesis rate (Pn). (**G**) Stomatal conductance (Gs). (**H**) Transpiration rate (Tr). (**I**) Water Use Efficiency (WUE). (**J**) Maximum photochemical efficiency of Photosystem II (Fv/Fm). (**K**) Non-photochemical quenching (Npq). (**L**) Photochemical quenching coefficient (qp). Error bars, mean ± SD. Each red dot represents a sample. The asterisks indicate a statistically significant difference (two-tailed Student’s *t*-test, * *p* < 0.05, and ** *p* < 0.01, *** *p* < 0.001, and **** *p* < 0.0001).

Plants respond to drought stress by adapting leaf morphology. Under drought conditions, leaves curl or curve to reduce transpiration. These morphological adjustments were more pronounced in the wild-type plants, where leaf length and width were significantly reduced compared to transgenic lines (Figure 4A–D). Under drought stress, the Pn, Gs, WUE, and qp values of *SiLEA5*-overexpressing lines were significantly higher, while Ci and Tr values were significantly lower than those in wild-type plants (Figure 4E–L). These results indicate that transgenic lines exhibit enhanced photosynthetic efficiency, effectively utilize water and CO_2_, improve drought resistance, and thrive in dry environments.

### 3.4. The SiLEA5 Enhances Plant Productivity

**Figure 5:**
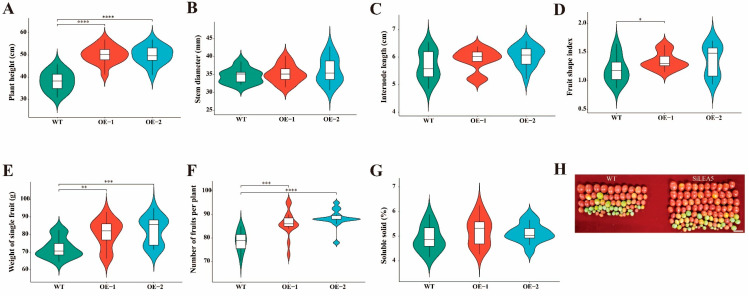
Statistics of growth indicators, yield, and quality of wild-type and transgenic lines. (**A**–**D**) Measurement results of tomato growth indicators. (**E**–**G**) Tomato yield and quality comparison. (**H**) Fruit morphology of wild-type and transgenic tomato. Error bars, mean ± SD. The asterisks indicate a statistically significant difference (two-tailed Student’s *t*-test, * *p* < 0.05, ** *p* < 0.01, *** *p* < 0.001, and **** *p* < 0.0001).

Crop production performance is a key indicator of productivity and economic viability. Statistical analysis showed that *SiLEA5* significantly increased plant height, with transgenic tomatoes averaging 11.83 cm taller than wild-type, highlighting *SiLEA5*’s role in regulating growth and development. Additionally, *SiLEA5* positively affected the fruit shape index, resulting in more uniform and moderately sized fruits. This improvement in fruit shape and fullness emphasizes *SiLEA5*’s role in fruit development (Figure 5A–H). Increased *SiLEA5* expression also enhanced plant yield, likely by promoting photosynthesis and respiration, thus boosting the supply of photosynthates and energy. Importantly, the higher expression of *SiLEA5* did not negatively affect tomato quality, underscoring its potential for yield improvement.

### 3.5. SiLEA5 Gene May Be Involved in ABA Metabolic Pathway

Cis-acting element analysis revealed that the promoter region of the *SiLEA5* gene contains elements responsive to drought stress and ABA, suggesting that this gene may play a significant role in drought response and ABA signal transduction in plants. To test this hypothesis, *SiLEA5* gene expression levels in tomato leaves were measured after 20 days of drought treatment and 48 h following 100 μM ABA treatment. The results showed that both drought and ABA treatments significantly induced the upregulation of *SiLEA5* expression (Supplementary Figure S1), indicating that *SiLEA5* might contribute to the plant’s response to ABA-dependent drought stress.

**Figure 6:**
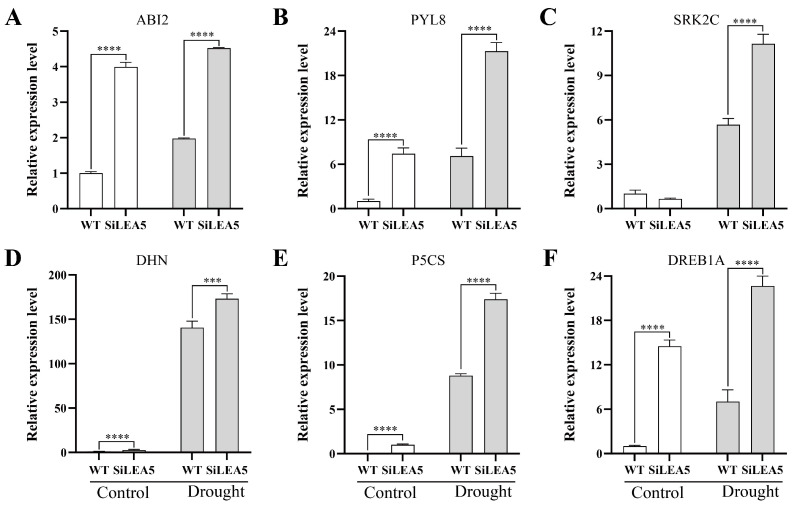
Expression analysis of relevant genes in transgenic and wild-type tomato. (**A**) Relative expression levels of the *ABI2* gene. (**B**) Relative expression level of *PYL8* gene. (**C**) Relative expression level of *SRK2C* gene. (**D**) Relative expression level of *DHN* gene. (**E**) Relative expression level of *P5CS* gene. (**F**) Relative expression level of *DREB1A* gene. Three independent biological replicates were performed (*n* = 3). Error bars, mean ± SD. The asterisks indicate a statistically significant difference (two-tailed Student’s *t*-test, *** *p* < 0.001, and **** *p* < 0.0001).

AB12 functions as a transcription factor that regulates ABA response elements, while PYL8 and SRK2C serve as ABA receptors and protein kinases, respectively, involved in the perception and transmission of ABA signals. Together, these components play key roles in ABA signaling. To further elucidate the molecular mechanism by which overexpression of *SiLEA5* enhances drought resistance in tomatoes, qRT-PCR was used to analyze the expression of stress-related genes under drought conditions. As shown in Figure 6, the expression levels of *ABI2*, *PYL8*, and *DREB1A* in transgenic lines were significantly higher than in WT under non-stress conditions. After drought treatment, the expression levels of ABA signaling pathway-related genes, including *ABI2*, *PYL8*, and *SRK2C*, were significantly elevated in transgenic lines compared to WT. These findings suggest that the *SiLEA5* gene may enhance drought resistance in transgenic tomatoes by activating the expression of ABA signaling pathway-related genes. Under drought stress, the expression of the *P5CS* gene in transgenic lines was significantly upregulated, resulting in increased activity of key enzymes in the proline biosynthesis pathway. This, in turn, led to a substantial increase in proline content, consistent with the physiological response. The proteins encoded by the *DHN* gene family maintain the structural integrity of cells by forming protective protein networks. Under drought stress, the expressions of *DHN* genes were significantly upregulated. Notably, the expression of the *DHN* gene in transgenic lines increased 170-fold under non-stress conditions and was strongly correlated with drought stress. These results indicate that the *DHN* gene plays a crucial role in the plant’s drought response, and its significant upregulation may be a key mechanism for plant adaptation to drought. DREB1A, a transcription factor, activates downstream genes such as *COR*, enhancing plant tolerance to stress. This study found that under drought stress, the expression level of the *DREB1A* gene in transgenic lines was 3.2 times higher than in WT. After drought treatment, the expression of *PYL8*, *SRK2C*, *DHN*, *P5CS*, and *DREB1A* in transgenic lines was significantly higher than in non-stress conditions. These findings suggest that overexpression of the *SiLEA5* gene may enhance the transcriptional activity of these genes, improving the drought stress response and playing a central role in plant stress adaptation.

## 4. Discussions and Conclusions

LEA proteins are known for their disordered structural characteristics, which provide flexibility and enable interactions with various proteins, RNAs, and other biomolecules, thereby performing diverse biological functions [3]. In this study, we conducted a detailed analysis of the promoter region of the *SiLEA5* gene from *Saussurea involucrata* and identified cis-regulatory elements associated with drought stress and ABA responsiveness. These findings suggest that *SiLEA5* may directly participate in ABA-mediated signal transduction and stress responses. Further analysis revealed that *SiLEA5* expression is significantly upregulated after ABA and drought treatment, indicating dual regulation by both the ABA signaling pathway and drought stress. This dual regulatory mechanism enhances the rapid response ability of the *SiLEA5* gene to drought stress.

The LEA protein family plays a critical role in plant stress responses, serving as key protective factors that help plants adapt to and survive under stress conditions [26,27]. In our previous study, we demonstrated that *SiLEA5* enhances cold tolerance in tomato by regulating osmotic substances and antioxidant enzyme activities at −2 °C [19]. Proline, a vital osmoprotectant in plants, helps maintain cell water potential, stabilize cell membrane structures, and strengthen antioxidant defenses, thereby significantly reducing water loss and protecting cells from oxidative damage [28]. Additionally, proline interacts with endogenous enzymes and proteins, further enhancing plant biochemical stability under stress [29]. In this study, we found that under drought stress, transgenic tomatoes accumulated significantly more proline, and the key enzyme involved in proline synthesis, P5CS, was significantly upregulated under drought conditions, reaching levels ten times higher than those under non-stress conditions. This result is consistent with our physiological and biochemical data. Batool et al.’s research on rice further supports the role of proline in helping cells cope with stress [1]. Additionally, Du et al. demonstrated that *TaERF87* regulates the expression of the proline biosynthesis gene (*TaP5CS1*) in cooperation with the bHLH transcription factor *TaAKS1* to promote proline accumulation and enhance drought tolerance in wheat [5]. Therefore, *SiLEA5* may enhance the osmotic adjustment capacity and antioxidant defenses of transgenic tomatoes by regulating the expression of the *P5CS* gene, thereby improving the plants’ tolerance to drought stress.

In this study, the overexpression of the *SiLEA5* gene significantly enhanced the physiological adaptation and productivity of tomatoes under drought stress. Overexpression of *SiLEA5* not only promoted plant growth but also improved water physiology, as evidenced by a reduced leaf water loss rate and increased root vitality. These findings are consistent with research showing that LEA proteins improve drought tolerance by maintaining cellular water balance and protecting membrane integrity [13]. This ability to retain water is crucial for sustaining photosynthesis and water use efficiency under drought conditions [30]. Furthermore, we found that transgenic tomatoes exhibited higher photosynthetic efficiency and water use efficiency under drought stress. This may be due to *SiLEA5* optimizing stomatal behavior, thereby enhancing both photosynthesis and water utilization. This regulatory effect is essential for plants to efficiently use water and light energy in water-limited environments, which directly impacts crop productivity [31]. Ultimately, the overexpression of *SiLEA5* increased tomato yield, which has significant implications for enhancing agricultural economic benefits and sustainable development [32]. These results demonstrate that *SiLEA5* enhances tomato productivity and drought tolerance by regulating water physiology, root vitality, and photosynthesis, providing valuable scientific insights for further research into LEA protein function and regulatory mechanisms in plant stress responses.

LEA proteins play a key role in plant responses to drought and other stressors. Beyond regulating osmotic balance and protecting cell membranes [33], LEA proteins may also influence water distribution by modulating stomatal function [34]. Under water stress, stomatal regulation is crucial for plant water use efficiency and drought tolerance [35]. This study suggests that overexpression of *SiLEA5* may indirectly reduce excessive stomatal opening by regulating cell hydration and water balance, thus reducing transpiration losses and improving water retention capacity [36,37]. This hypothesis is supported by previous studies, which suggest that LEA proteins may be involved in stomatal closure regulation by affecting cell hydration [38], helping plants more effectively control water loss under stress [39]. Additionally, under drought and ABA treatment, transgenic tomatoes showed significantly lower stomatal aperture and density compared to WT, further supporting the potential role of LEA proteins in stomatal regulation. However, further work utilizing gene editing techniques is needed to validate the specific role of *SiLEA5* in stomatal regulation.

Plants facing osmotic stress rely on a complex signaling network involving multiple pathways that work synergistically, with the ABA signaling pathway playing a central role in stress responses [40,41]. In this study, we observed significant upregulation of ABA-related genes in transgenic tomatoes under drought stress, suggesting that *SiLEA5* may enhance drought tolerance by activating the ABA signaling pathway. Further analysis revealed that *SiLEA5* promotes the accumulation of DHN and DREB1A proteins through the ABA pathway. These proteins play protective roles under stress by maintaining cellular stability and shielding plants from oxidative damage caused by reactive oxygen species [42,43]. However, plant adaptation and response to stress involve a complex network of signaling pathways [44]. Previous studies have shown that under drought and salt stress, the interaction between the ABA and ethylene signaling pathways works synergistically to close stomata and reduce water loss [45,46]. Additionally, there is an antagonistic relationship between the ABA and gibberellin (GA) signaling pathways, which plays a critical regulatory role in plant growth and stress responses [47,48]. Although this study has some limitations regarding the interaction mechanisms between these signaling pathways, our findings open new directions for future research on the *SiLEA5* gene and may provide potential molecular targets for the genetic improvement of drought tolerance in crops.

**Figure 7:**
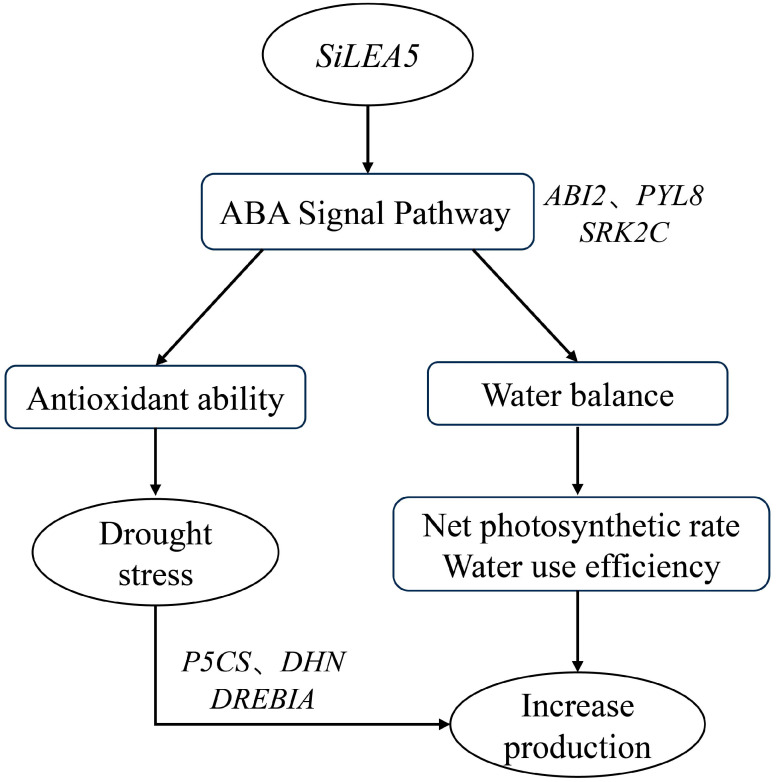
Schematic representation of the role of *SiLEA5* gene in drought stress regulation in tomatoes.

In conclusion, the *SiLEA5* gene enhances drought tolerance in tomato by participating in the ABA signaling pathway (Figure 7). Expression of *SiLEA5* not only improves water balance in plants but also strengthens antioxidant capacity, reducing oxidative damage under drought stress. Furthermore, it confers significant advantages in net photosynthetic rate and yield. These findings suggest that *SiLEA5* is a promising target for improving drought tolerance in tomatoes and other crops. This study provides essential theoretical support for the development of drought-resistant crop varieties, with potential implications for enhancing crop resilience to environmental stress and advancing agricultural productivity.

## Figures and Tables

**Table 1:** Cis-element prediction of the SiLEA5 promoter.

Name of Element	Core Sequence	Number	Biological Function
ARE	AAACCA	1	Cis-acting regulatory element essential for the anaerobic induction
WUN-motif	AAATTACT	2	Wound-responsive element
TGA-element	AACGAC	1	Auxin-responsive element
ABRE	ACGTG	2	Cis-acting element involved in the abscisic acid responsiveness
Box-4	ATTAAT	3	Part of a conserved DNA module involved in light responsiveness
TC-rich repeats	ATTCTCTAAC	1	Cis-acting element involved in defense and stress responsiveness
MBS	CAACTG	1	MYB binding site involved in drought inducibility
CAAT-box	CAAT(T)	13	Common cis-acting elements in promoter and enhancer regions
TCA-element	CCATCTTTTT	1	Cis-acting element involved in salicylic acid responsiveness
CGTCA-motif	CGTCA	1	Cis-acting regulatory element involved in the MeJ-responsiveness
TATA-box	TATA	17	Core promoter element around −30 of transcription start
TATC-box	TATCCCA	1	Cis-acting element involved in gibberellin responsiveness
W-box	TTGACC	2	WRKY transcription factor binding site

## Data Availability

The original contributions presented in this study are included in the article/Supplementary Materials. Further inquiries can be directed to the corresponding authors.

## Supplementary Materials

The following supporting information can be downloaded at https://www.mdpi.com/article/10.3390/foods13223641/s1, Figure S1: Real-time PCR was used to detect wild-type and transgenic tomatoes; Table S1: Primers used in qRT-PCR analysis.

## References

[1] Batool T., Ali S., Seleiman M.F., Naveed N.H., Ali A., Ahmed K., Abid M., Rizwan M., Shahid M.R., Alotaibi M. (2020). Plant Growth Promoting Rhizobacteria Alleviates Drought Stress in Potato in Response to Suppressive Oxidative Stress and Antioxidant Enzymes Activities. Sci. Rep..

[2] Abdul Aziz M., Sabeem M., Mullath S.K., Brini F., Masmoudi K. (2021). Plant Group II LEA Proteins: Intrinsically Disordered Structure for Multiple Functions in Response to Environmental Stresses. Biomolecules.

[3] Chen J., Li N., Wang X., Meng X., Cui X., Chen Z., Ren H., Ma J., Liu H. (2021). Late Embryogenesis Abundant (LEA) Gene Family in *Salvia miltiorrhiza*: Identification, Expression Analysis, and Response to Drought Stress. Plant Signal. Behav..

[4] Chong L., Xu R., Huang P., Guo P., Zhu M., Du H., Sun X., Ku L., Zhu J.-K., Zhu Y. (2022). The Tomato OST1–VOZ1 Module Regulates Drought-Mediated Flowering. Plant Cell.

[5] Du L., Huang X., Ding L., Wang Z., Tang D., Chen B., Ao L., Liu Y., Kang Z., Mao H. (2023). TaERF87 and TaAKS1 Synergistically Regulate TaP5CS1/TaP5CR1-Mediated Proline Biosynthesis to Enhance Drought Tolerance in Wheat. New Phytol..

[6] Footitt S., Hambidge A.J., Finch-Savage W.E. (2021). Changes in Phenological Events in Response to a Global Warming Scenario Reveal Greater Adaptability of Winter Annual Compared with Summer Annual Arabidopsis Ecotypes. Ann. Bot..

[7] Giordani T., Natali L., D’Ercole A., Pugliesi C., Fambrini M., Vernieri P., Vitagliano C., Cavallini A. (1999). Expression of a Dehydrin Gene during Embryo Development and Drought Stress in ABA-Deficient Mutants of Sunflower (*Helianthus annuus* L.). Plant Mol. Biol..

[8] Zhao Y., Hao Y., Dong Z., Tang W., Wang X., Li J., Wang L., Hu Y., Fang L., Guan X. (2023). Identification and Expression Analysis of LEA Gene Family Members in Pepper (*Capsicum annuum* L.). FEBS Open Bio..

[9] Geng W., Wang Y., Zhang J., Liu Z., Chen X., Qin L., Yang L., Tang H. (2022). Genome-Wide Identification and Expression Analyses of Late Embryogenesis Abundant (LEA) Gene Family in Tobacco (*Nicotiana tabacum* L.) Reveal Their Function in Abiotic Stress Responses. Gene.

[10] Hajihashemi S., Brestic M., Landi M., Skalicky M. (2020). Resistance of Fritillaria Imperialis to Freezing Stress through Gene Expression, Osmotic Adjustment and Antioxidants. Sci. Rep..

[11] Fan J., Zhang Y., Sun H., Duan R., Jiang Y., Wang X., Sun Y., Luo Z., Wang P., Guan S. (2024). Overexpression of Soybean *GmDHN9* Gene Enhances Drought Resistance of Transgenic *Arabidopsis*. GM Crops Food.

[12] Onyemaobi O., Sangma H., Garg G., Wallace X., Kleven S., Suwanchaikasem P., Roessner U., Dolferus R. (2021). Reproductive Stage Drought Tolerance in Wheat: Importance of Stomatal Conductance and Plant Growth Regulators. Genes.

[13] Razi K., Muneer S. (2021). Drought Stress-Induced Physiological Mechanisms, Signaling Pathways and Molecular Response of Chloroplasts in Common Vegetable Crops. Crit. Rev. Biotechnol..

[14] Jin X., Cao D., Wang Z., Ma L., Tian K., Liu Y., Gong Z., Zhu X., Jiang C., Li Y. (2019). Genome-Wide Identification and Expression Analyses of the LEA Protein Gene Family in Tea Plant Reveal Their Involvement in Seed Development and Abiotic Stress Responses. Sci. Rep..

[15] Jan R., Khan M.-A., Asaf S., Lubna, Waqas M., Park J.-R., Asif S., Kim N., Lee I.-J., Kim K.-M. (2022). Drought and UV Radiation Stress Tolerance in Rice Is Improved by Overaccumulation of Non-Enzymatic Antioxidant Flavonoids. Antioxidants.

[16] Ju H., Li D., Li D., Yang X., Liu Y. (2021). Overexpression of ZmDHN11 Could Enhance Transgenic Yeast and Tobacco Tolerance to Osmotic Stress. Plant Cell Rep..

[17] Ju Y., Yue X., Min Z., Wang X., Fang Y., Zhang J. (2020). VvNAC17, a Novel Stress-Responsive Grapevine (*Vitis vinifera* L.) NAC Transcription Factor, Increases Sensitivity to Abscisic Acid and Enhances Salinity, Freezing, and Drought Tolerance in Transgenic Arabidopsis. Plant Physiol. Biochem..

[18] Wang G., Xu X., Gao Z., Liu T., Li Y., Hou X. (2022). Genome-Wide Identification of LEA Gene Family and Cold Response Mechanism of *BcLEA4-7* and *BcLEA4-18* in Non-Heading Chinese Cabbage [*Brassica campestris* (Syn. Brassica rapa) Ssp. chinensis]. Plant Sci..

[19] Liu X.-Y., Xia W.-W., Zhang X.-L., Li A.-W., Qin J., Sun H.-L., Li J., Zhu J.-B. (2022). Overexpression of the *SiLEA5* Gene in *Saussurea involucrata* Increases the Low-Temperature Tolerance of Transgenic Tomatoes. Horticulturae.

[20] Xin H., Li Q., Wang S., Zhang Z., Wu X., Liu R., Zhu J., Li J. (2023). *Saussurea involucrate* PIP2;4 Improves Growth and Drought Tolerance in *Nicotiana tabacum* by Increasing Stomatal Density and Sensitivity. Plant Sci..

[21] Zhang C., Niu D., Zhang L., Li X., Fu H. (2022). Plant Functional Traits Shape Growth Rate for Xerophytic Shrubs. Plant Biol. J..

[22] Richter A.K., Frossard E., Brunner I. (2007). Polyphenols in the Woody Roots of Norway Spruce and European Beech Reduce TTC. Tree Physiol..

[23] Wu Y., Yang H., Yang H., Zhang C., Lyu L., Li W., Wu W. (2022). A Physiological and Metabolomic Analysis Reveals the Effect of Shading Intensity on Blueberry Fruit Quality. Food Chem. X.

[24] Liu X., Xia W., Zhang D., Li A., Li J., Zhu J. (2023). Cold Tolerance Gene SiLEA B19.3 of *Saussurea involucrate* Increases the Yield of Transgenic Tomato. S. Afr. J. Bot..

[25] Liu X., Li A., Wang S., Lan C., Wang Y., Li J., Zhu J. (2022). Overexpression of *Pyrus sinkiangensis* HAT5 Enhances Drought and Salt Tolerance, and Low-Temperature Sensitivity in Transgenic Tomato. Front. Plant Sci..

[26] Li N., Zhang S., Liang Y., Qi Y., Chen J., Zhu W., Zhang L. (2018). Label-Free Quantitative Proteomic Analysis of Drought Stress-Responsive Late Embryogenesis Abundant Proteins in the Seedling Leaves of Two Wheat (*Triticum aestivum* L.) Genotypes. J. Proteom..

[27] Kwon E., Basnet P., Roy N.S., Kim J.-H., Heo K., Park K.-C., Um T., Kim N.-S., Choi I.-Y. (2021). Identification of Resurrection Genes from the Transcriptome of Dehydrated and Rehydrated *Selaginella tamariscina*. Plant Signal. Behav..

[28] Liu X., Wang L., Zhang X., Li A., Xia W., Lin C., Li J., Zhu J. (2023). Expression of the *Pyrus sinkiangensis* HD-Zip Ι Transcription Factor *PsHB7* and *PsHB12* in Hybrid *Broussonetia papyrifera* Regulates Its Natural Overwintering. Environ. Exp. Bot..

[29] Nong Q., Malviya M.K., Solanki M.K., Solanki A.C., Lin L., Xie J., Mo Z., Wang Z., Song X.-P., Huang X. (2022). Sugarcane Root Transcriptome Analysis Revealed the Role of Plant Hormones in the Colonization of an Endophytic Diazotroph. Front. Microbiol..

[30] Xie J., Yin G., Ma D., Chen R., Zhao W., Xie Q., Wang C., Lin S., Yuan W. (2024). Climatic Limitations on Grassland Photosynthesis over the Tibetan Plateau Shifted from Temperature to Water. Sci. Total Environ..

[31] Lawson T., Vialet-Chabrand S. (2019). Speedy Stomata, Photosynthesis and Plant Water Use Efficiency. New Phytol..

[32] Thudi M., Palakurthi R., Schnable J.C., Chitikineni A., Dreisigacker S., Mace E., Srivastava R.K., Satyavathi C.T., Odeny D., Tiwari V.K. (2021). Genomic Resources in Plant Breeding for Sustainable Agriculture. J. Plant Physiol..

[33] Murvai N., Kalmar L., Szabo B., Schad E., Micsonai A., Kardos J., Buday L., Han K.-H., Tompa P., Tantos A. (2021). Cellular Chaperone Function of Intrinsically Disordered Dehydrin ERD14. IJMS.

[34] Zhao Y., Fu X., Zou Z. (2024). Insights into Genes Encoding LEA_1 Domain-Containing Proteins in Cyperus Esculentus, a Desiccation-Tolerant Tuber Plant. Plants.

[35] Martin-StPaul N., Delzon S., Cochard H. (2017). Plant Resistance to Drought Depends on Timely Stomatal Closure. Ecol. Lett..

[36] Gao H., Cui J., Liu S., Wang S., Lian Y., Bai Y., Zhu T., Wu H., Wang Y., Yang S. (2022). Natural Variations of ZmSRO1d Modulate the Trade-off between Drought Resistance and Yield by Affecting ZmRBOHC-Mediated Stomatal ROS Production in Maize. Mol. Plant.

[37] Gray J., Dunn J. (2024). Optimizing Crop Plant Stomatal Density to Mitigate and Adapt to Climate Change. Cold Spring Harb. Perspect. Biol..

[38] López-Cordova A., Ramírez-Medina H., Silva-Martinez G.-A., González-Cruz L., Bernardino-Nicanor A., Huanca-Mamani W., Montero-Tavera V., Tovar-Aguilar A., Ramírez-Pimentel J.-G., Durán-Figueroa N.-V. (2021). LEA13 and LEA30 Are Involved in Tolerance to Water Stress and Stomata Density in Arabidopsis Thaliana. Plants.

[39] Muhammad Aslam M., Waseem M., Jakada B.H., Okal E.J., Lei Z., Saqib H.S.A., Yuan W., Xu W., Zhang Q. (2022). Mechanisms of Abscisic Acid-Mediated Drought Stress Responses in Plants. IJMS.

[40] Wang P., Zhao Y., Li Z., Hsu C.-C., Liu X., Fu L., Hou Y.-J., Du Y., Xie S., Zhang C. (2018). Reciprocal Regulation of the TOR Kinase and ABA Receptor Balances Plant Growth and Stress Response. Molecular Cell.

[41] Bulgakov V.P., Wu H.-C., Jinn T.-L. (2019). Coordination of ABA and Chaperone Signaling in Plant Stress Responses. Trends Plant Sci..

[42] Shibuya T., Itai R., Maeda M., Kitashiba H., Isuzugawa K., Kato K., Kanayama Y. (2020). Characterization of *PcLEA14*, a Group 5 Late Embryogenesis Abundant Protein Gene from Pear (*Pyrus communis*). Plants.

[43] Zhou M., Peng N., Yang C., Wang C. (2022). The Poplar (*Populus trichocarpa*) Dehydrin Gene PtrDHN-3 Enhances Tolerance to Salt Stress in Arabidopsis. Plants.

[44] Soltabayeva A., Dauletova N., Serik S., Sandybek M., Omondi J.O., Kurmanbayeva A., Srivastava S. (2022). Receptor-like Kinases (LRR-RLKs) in Response of Plants to Biotic and Abiotic Stresses. Plants.

[45] Agurla S., Gahir S., Munemasa S., Murata Y., Raghavendra A.S. (2018). Mechanism of Stomatal Closure in Plants Exposed to Drought and Cold Stress. Adv. Exp. Med. Biol..

[46] Brookbank B.P., Patel J., Gazzarrini S., Nambara E. (2021). Role of Basal ABA in Plant Growth and Development. Genes.

[47] Verma V., Ravindran P., Kumar P.P. (2016). Plant Hormone-Mediated Regulation of Stress Responses. BMC Plant Biol..

[48] Xie Z., Jin L., Sun Y., Zhan C., Tang S., Qin T., Liu N., Huang J. (2024). OsNAC120 Balances Plant Growth and Drought Tolerance by Integrating GA and ABA Signaling in Rice. Plant Commun..

