# Supplementary material for: Saussurea involucrata SiLEA5 Enhances Tolerance to Drought Stress in Solanum lycopersicum
Source: Foods. 2024 Nov 15;13(22):3641. doi: 10.3390/foods13223641 (PMC11594126; doi:10.3390/foods13223641)
Supplement: Supplementary file 1 [file foods-13-03641-s001.zip › foods-3289769-supplementary.pdf]

Supplementary Material

Supplementary Figure S1

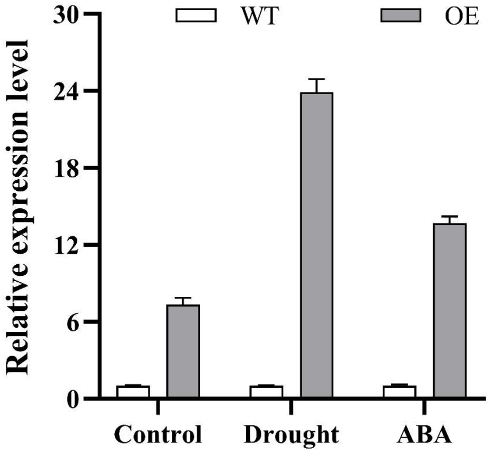

Supplemental Figure S1. Real-time PCR was used to detect wild−type and transgenic tomatoes.

Supplementary Table S1

Table S1. Primers used in qRT-PCR analysis

gene	Forward primer (5’→3’)	Reverse primer (5’→3’)
PYL8	CTTGGCATCAGAATCGTTGG	CCTCTTTAGTGTTCCCTTCAGG
DREB1A	AGCCAATCATTCTGACGAGG	GACTTCACAAACCCATTTTCCAG
ABI2	CCTAGTTTTAGCCAGTGACGG	CTTTCAGGCAGAGGGTTAGTC
DHN	GGCAATTTCATCTGAGTTGTCTG	TCTGTCCATCCTCTCCAATTTC
SRK2C	GTTGGAACTCCGGCCTATATC	TCAGGATCTTCAAACGGATACG
P5CS	ACGACCTGATGCACTTGTAC	ACTTACAGGAATTGCCGAGG
GAPDH	TAGCAAGGATGCTCCCATGTTCGT	AAAGGAGCAAGGCAGTTGGTTGTG
SiLEA5	GGTCAGATTTGGACGCTAGAG	GATACCCTTCAGTTCCCAGC
